# Environment-Induced Degradation of Shape Memory Alloys: Role of Alloying and Nature of Environment

**DOI:** 10.3390/ma16165660

**Published:** 2023-08-17

**Authors:** S. Santosh, W. B. Jefrin Harris, T. S. Srivatsan

**Affiliations:** 1 Department of Mechanical Engineering, Sri Sivasubramaniya Nadar College of Engineering, Chennai 603110, India; 2 Department of Mechanical Engineering, The University of Akron, Akron, OH 44325, USA; tss1@uakron.edu

**Keywords:** shape memory alloy, smart materials, environment-induced degradation, corrosion behavior

## Abstract

Shape memory effects coupled with superelasticity are the distinctive characteristics of shape memory alloys (SMAs), a type of metal. When these alloys are subject to thermomechanical processing, they have the inherent ability to react to stimuli, such as heat. As a result, these alloys have established their usefulness in a variety of fields and have in recent years been chosen for use in stents, sensors, actuators, and several other forms of life-saving medical equipment. When it comes to the shape memory materials, nickel–titanium (Ni-Ti) alloys are in the forefront and have been chosen for use in a spectrum of demanding applications. As shape memory alloys (SMAs) are chosen for use in critical environments, such as blood streams (arteries and veins), orthodontic applications, orthopedic implants, and high temperature surroundings, such as actuators in aircraft engines, the phenomenon of environment-induced degradation is of both interest and concern. Hence, the environment-induced degradation behavior of the shape memory alloys (SMAs) needs to be studied to find viable ways to improve their resistance to an aggressive environment. The degradation that occurs upon exposure to an aggressive environment is often referred to as corrosion. Environment-induced degradation, or corrosion, being an unavoidable factor, certain techniques can be used for the purpose of enhancing the degradation resistance of shape memory alloys (SMAs). In this paper, we present and discuss the specific role of microstructure and contribution of environment to the degradation behavior of shape memory alloys (SMAs) while concurrently providing methods to resist both the development and growth of the degradation caused by the environment.

## 1. Introduction

A shape memory alloy (SMA) has few distinctive qualities to essentially include shape memory effect and superelasticity [1], which makes the alloy desirable for selection and use in biomedical and several other intricate applications. The shape memory alloys (SMAs), such as nickel–titanium (NiTi), have been chosen for use in both orthopedic applications and orthodontic applications for many years [2]. Through the years, these sectors have seen particularly noticeable improvements. The shape memory alloy is gradually replacing other materials as the preferred option for use as, e.g., filters, baskets, self-expansion stents, support system grafts, and a few other devices, which find use in minimally invasive interventional treatments. The possible impact on biocompatibility is still a source of concern. Also, the contradictory environment-induced degradation resistance, or corrosion, serves to complicate this issue. Nickel, which is necessary for both nourishment and bodily function [3], is found in human tissue in amounts of about 0.1 ppm. A higher nickel concentration that could leak out of the implant material could cause negative allergies, be toxic, and even promote cancerous reactions. Furthermore, NiTi and 316L stainless steel [4] MP35N (35 weight percent nickel) is another widely chosen and used high nickel-containing alloy that demonstrates strong biocompatibility and is often chosen for use as an implant for orthopedic applications spanning cardiovascular and orthodontic uses. Like the other non-noble implant metals, the environment-induced degradation resistance of a nickel–titanium (NiTi) alloy largely depends on the existence or presence of a passive coating on the surface. No general degradation upon exposure to aggressive environmental issues has to be anticipated so long as the passive film is in good condition. However, it should be kept in mind that the release of certain ions can also happen in the passive state. Additionally, the passive film could easily break down during both surgery and service life. Hence, the most concerning factor for an effective use of the shape memory alloys (SMAs) is the degradation that occurs upon exposure to an aggressive environment and there exists a need to work towards enabling an enhancement and/or improvement of the resistance to environment-induced degradation properties. In the following sections, a few ways to increase the resistance to environment-induced degradation of the different shape memory alloys (SMAs), along with adequate discussion of the specifics pertinent to the degradation response or corrosion behavior of a few shapes memory alloys (SMAs), are provided.

## 2. Environment-Induced Degradation and Its Mechanism

**Figure 1:**
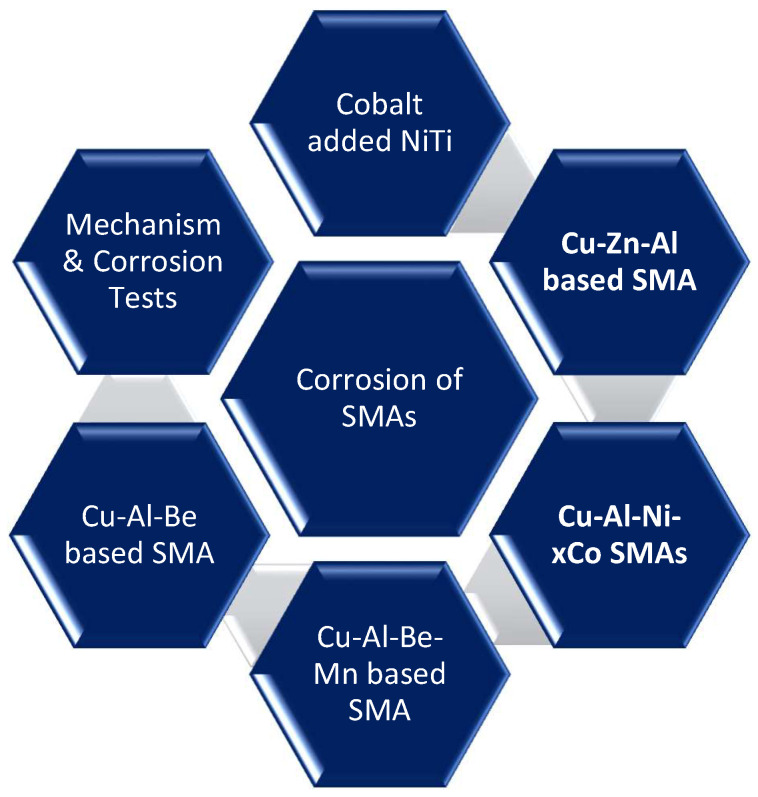
Environment-induced degradation or corrosion of various shape memory alloys (SMAs).

Environment-induced degradation, referred to as corrosion, is defined as the chemical–physical metal–environment interactions that often result in an observable modification to the properties of the chosen metal and the resultant deficit in the functioning of the metal, the medium, or the system, which essentially includes both the metal and the medium. When a substance is at its peak perfection, it is desirable to shield it from any adverse activity arising from being influenced and/or affected by an external action resulting from exposure to an aggressive environment. The best method to safeguard metal surfaces is to introduce coatings. Like mortality, the occurrence of environment-induced degradation or corrosion is unfortunate, and every effort is made to prevent it from occurring. Essentially, it is a phenomenon that we must learn to not only manage but to eventually conquer [4,5]. A system’s decreased Gibbs free energy is the main contributing factor to degradation by corrosion. As a result, the specific metal has a significant inclination to revert to its original lower energy state. Environment-induced degradation or corrosion is the term given for this return to the original state and is shown in Figure 1. Although the occurrence of degradation by corrosion is inevitable, it can be slowed down to reach equilibrium. When considering a coating for the purpose of preventing environment-induced degradation, i.e., corrosion, it must provide a strong physical barrier that prevents the aggressive elements in the environment from reaching the metal surface. The degradation by corrosion of metals and alloys can safely be a complex issue that has plagued human beings for a considerable amount of time [5]. Due to their exceptional corrosion resistance, nickel-based alloys are frequently utilized in industry under challenging conditions. As a result, alloys based on nickel are commonly used in corrosive applications [6]. Hence, it is more important to study the behavior of these functional alloys under corrosive environment, as shown below.

The mechanism illustrated above implies that mass loss with immersion time exhibits an erratic development at first, followed by a fast increase in the middle and, eventually, a tendency towards stability. This shows that the solid-state diffusion growth mechanism, which provides the protective behavior, is under control of the growth of corrosion products and that the corrosion products have a restricted rate of dissolution in molten salt.

## 3. Various Corrosion Resistance Tests and Their Procedures

### 3.1. Various Test Solutions Used

**Figure 2:**
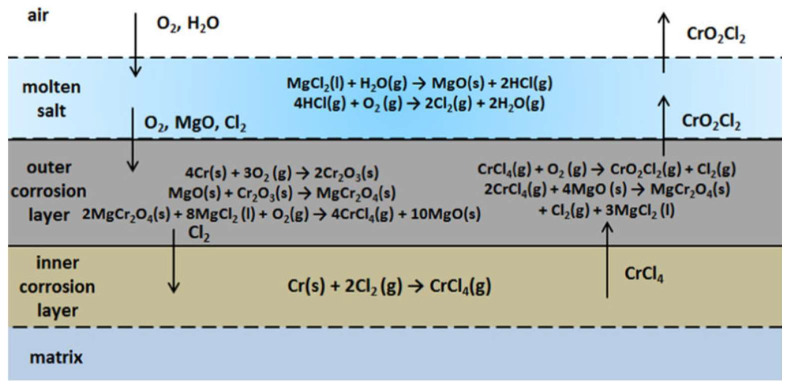
Corrosion mechanism of Ni-based alloy in NaCl-CaCl_2_-MgCl_2_. Environment [5] (reused with permission from Elsevier).

**Figure 3:**
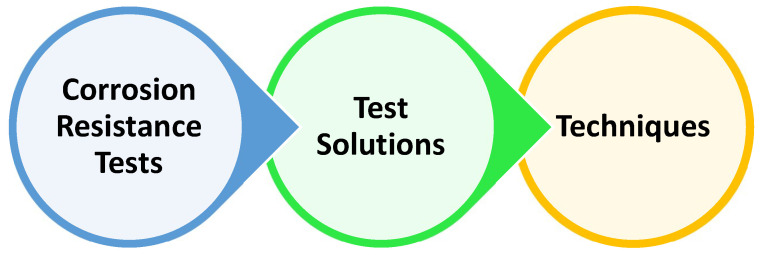
Depiction of the corrosion test and its procedure.

**Figure 4:**
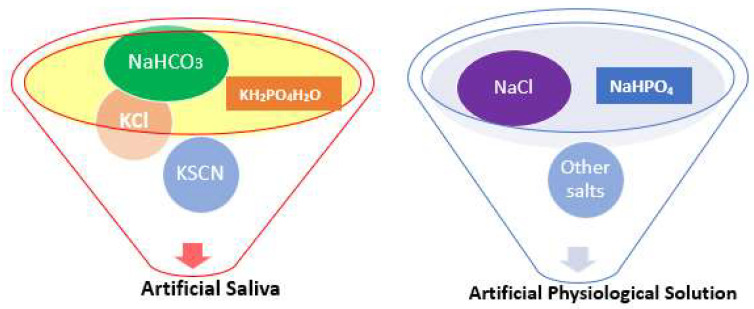
Schematic composition of the solutions used.

The solutions used in in vitro electrochemical testing are shown in Figure 2. The solution temperature in potentiostatic and potentiodynamic scratch tests were set to 4 °C. Modified ASTM tests were carried out at a temperature of 37 °C [7]. The two rising temperature tests were the following: (i) crevice temperature tests and (ii) potentiostatic critical pitting test. The corrosion test procedure and composition is presented in Figure 3 and Figure 4, respectively.

### 3.2. Techniques

**Figure 5:**
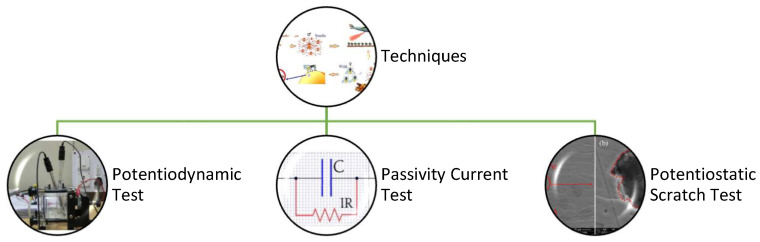
Types of tests to study environment-induced degradation.

The various types of tests used are shown in Figure 5. Polishing of the test specimens was performed using 1-micron diamond paste for both the potentiodynamic tests and the potentiostatic scratch tests. The rest of the test specimens were mechanically ground using a 600-mesh wheel or 600-mesh emery paper [8,9].

### 3.3. The Potentiodynamic Test

The ASTM G5 practice-recommended experimental setup was used to conduct the cyclic potentiodynamic tests. The ASTM G61 practice was followed for flat specimens using a specimen holder and a scan rate of 600 mV/h and starting potential of 750 mV [10,11,12]. The direction of scanning was interchanged when the current density of the anode attained 100 µA/cm^2^.

### 3.4. Passivity Current Test

Potentiostatic experiments were conducted at 100 mV in de-aerated settings versus standard calomel electrode (SCE). The current levels were periodically recorded for various times to both evaluate and establish the passivity current. Preparation of the cylindrical test specimens was very much in conformance with details specified in the standard ASTM G5.

### 3.5. Potentiostatic Scratch Test

Additionally, for the flat test specimen, the electrode assembly described in ASTM G61 standard practice was used. The scratch test approach, which was first presented by Pessal and Liu [13,14,15,16], enables an identification of the potential value at which repair of the injured passive film can be prevented. This technique involves delicately scratching the metal surface using a diamond tip while concurrently using a potentiostat to adjust the potential, with the initial potential being in the passive area. Further scratching is performed at a higher potential level should the scratched region re-passivate [17]. Therefore, it is possible to calculate the potential at which the chosen test specimen will not re-passivate.

## 4. The Copper-Aluminum-Beryllium (Cu-Al-Be)-Based Shape Memory Alloy

**Figure 6:**
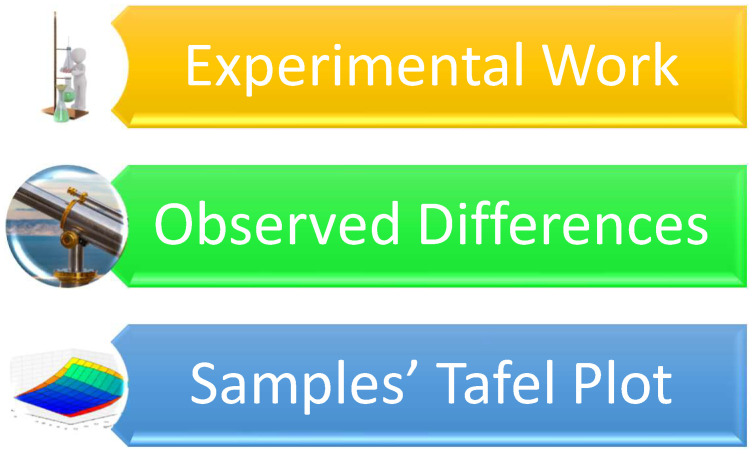
Layout of the copper-aluminum-beryllium (Cu-Al-Be)-based shape memory alloy (SMA).

The copper-based shape memory alloys are less expensive, good to make, and, importantly, easy to work with. However, because they are both fragile and tedious to manufacture, the copper-based shape memory alloys (SMAs) cannot be readily chosen for use. Grain refining has often been used in an attempt to increase the resilience of multicrystalline copper-based shape memory alloys with varying degrees of success [18,19]. However, recent research studies have revealed that the copper-aluminium-manganese shape memory alloys (SMAs) have noticeably better ductility due to the presence of the progenitor phase, which essentially has an L21 morphology with less degree order. Shape memory alloys (SMAs) have been chosen for use in a variety of applications to esentially include the following: (i) valves, (ii) pipes, and (iii) fasteners. Shape memory alloys (SMAs) are often exposed to aggressive environments, spanning both aqueous and gaseous environments, during service in many applications. Degradation induced by the environment, referred to as corrosion, is primarily responsible for well over 75 percent of the gradual decay and/or deterioration experienced by the material, resulting in the occurrence of failure due to changes in properties while in use or service in a variety of applications [20,21]. Aschematic of the test is shown in Figure 6.

### 4.1. Experimental Work

**Figure 7:**
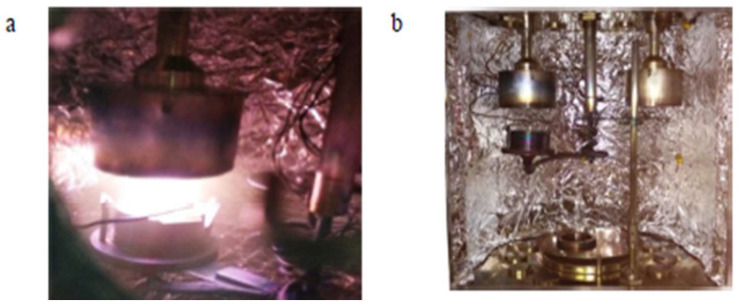
(**a**) Surface exposed to plasma arc and (**b**) apparatus used for sputtering [21]. (Reused with permission from Elsevier).

A composition of 11.5 weight percent aluminum, 0.44 weight percent beryllium (Be), and the remaining copper was chosen to complete the current research study as shown in Figure 7. Small bits of copper (Cu), aluminum (Al), and beryllium (Be), each weighing about 100 g for the chosen composition, were taken and then converted to the molten state in an induction furnace. The molten alloy was given time to harden in a permanent mold that measured 150 mm × 100 mm × 5 mm [22,23,24]. The ingots that were produced were subsequently homogenized. Subsequently, a spectrophotometer that measures plasma-optical emission was used to analyze composition of the alloy. Identical samples were rolled to a thickness of about 1 mm at 900 °C. The test samples were then subjected to the following treatment:

(i) Betatized for a full 30 min at 900 °C;
(ii) Step-cooled in water that was boiling (100 °C);
(iii) Finally cooled in a tub containing water at approximately 30 °C [25].

An optical microscope was used to examine the microstructure and morphology of the martensite that was produced. A bend test was used to check the samples produced for the shape memory effect. The coating for the reference sample was carried out utilizing the sputtering technique. The apparatus used for sputtering created a plasma phase while concurrently maintaining a deposition pressure of 6 × 10^−3^ millibar and a base pressure of 1 × 10^−5^ millibar. The sample essentially served as the anode, while titanium served as the cathode, with their separation being a distance of 45 mm. With a current of 120 amps and a DC bias voltage of 0.6 kV, the process was permitted to operate for a full 25 min [26,27,28,29].

The samples were chopped into rectangular shapes that were then utilized for conducting the corrosion test. Fresh water and Hank’s solution were both chosen to be the aggressive aqueous media. A potentiostat was connected to the anode and opposite terminal after being placed in an appropriate holder [30]. During the test, a graphite rod served as the counter cathode and a wet calomel cathode as the reference anode. The product regulated both the environment-induced degradation process and its impact upon exposure to an aggressive environment. This helped in the following:

(a) Maintaining the desired voltage in the circuit;
(b) Estimating the characteristics of the current;
(c) Displaying the yield as a voltage (E) versus current (I) plot.

By using a voltage range between −2.4 V and 3.0 V and an output rate of 5.0 mV/s, anodic polarization curves of the chosen alloy were obtained [31].

### 4.2. Observed Differences

The shape memory effect (SME) of the chosen alloy that was identified using a bend test is summarized in Table 1. Without a coating, the shape memory alloys revealed an appreciable shape memory effect of 82 percent and 89 percent. However, with a coating on the shape memory alloy, the shape memory effect (SME) was 66 percent and 74 percent [32,33]. The amount of martensite that was changed and subsequently reverted to austenite determined how much strain could be recovered due to the shape memory effect (SME). The decrease in shape memory effect (SME) was made possible by the gradual growth of the intermetallic oxides, i.e., titanium dioxide (TiO_2_). The stress caused by the presence of the titanium layer and its thermal expansion coefficient on the shape memory alloy (SMA) was a contributing factor to the observable decline of the shape memory effect (SME).

**Figure 8:**
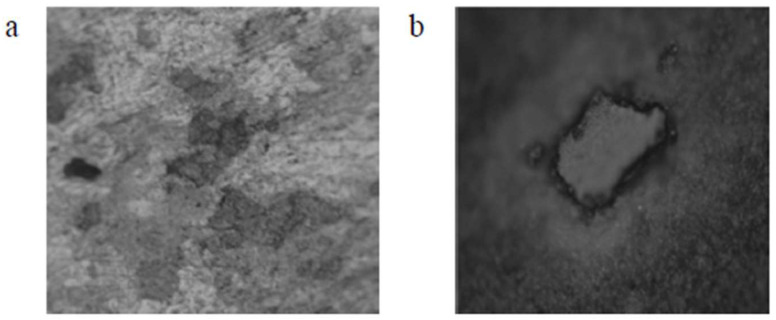
CAB1 sample optical micrograph at 20X resolution. (**a**) Evidence of degradation resulting from exposure to fresh water (H_2_O) (50 nm). (**b**) Evidence of degradation resulting from exposure to Hank’s solution (50 nm) and surface morphology of the chosen shape memory alloy (CAB1) [21] (reused with permission from Elsevier).

**Figure 9:**
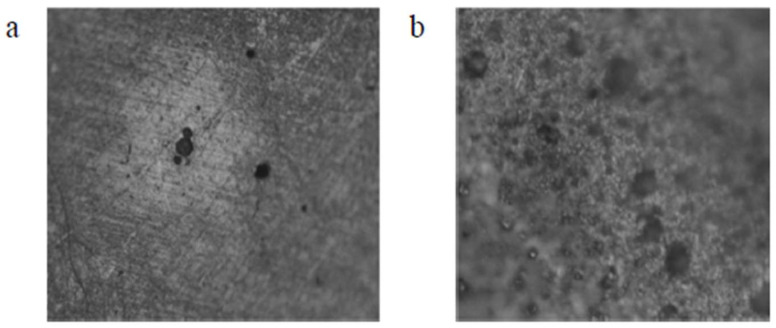
CAB3 alloy sample’s optical micrograph at 20X. (**a**) Evidence of degradation resulting from exposure to pure H_2_O (50 nm). (**b**) Evidence of degradation resulting from exposure to Hank’s solution. (50 nm); surface morphology of the shape memory alloy (SMA: CAB3) [21] (reused with permission from Elsevier).

For the combination tests considering two consuming media, the rate of degradation induced or caused by exposure to an aggressive environment was determined. E_(corr)_ provided an indication of the starting point of the detachable layer’s depletion, whereas E provided an indication of the point at which the detached layer had completely drained and thereby caused the formation and presence of pits in the chosen material as shown in Figure 8 and Figure 9 [28,29,30,31,32,33,34,35]. Upon exposure to an aggressive aqueous environment, it was found that covering the copper-based alloys with titanium increased their corrosion resistance. The gradual decomposition of the admixture caused the release of cuprous ions that were harmful. The beryllium–copper alloy resisted marine fouling and was found to be suitable for use in marine applications [36,37,38].

### 4.3. The Samples’ Tafel Plot

**Figure 10:**
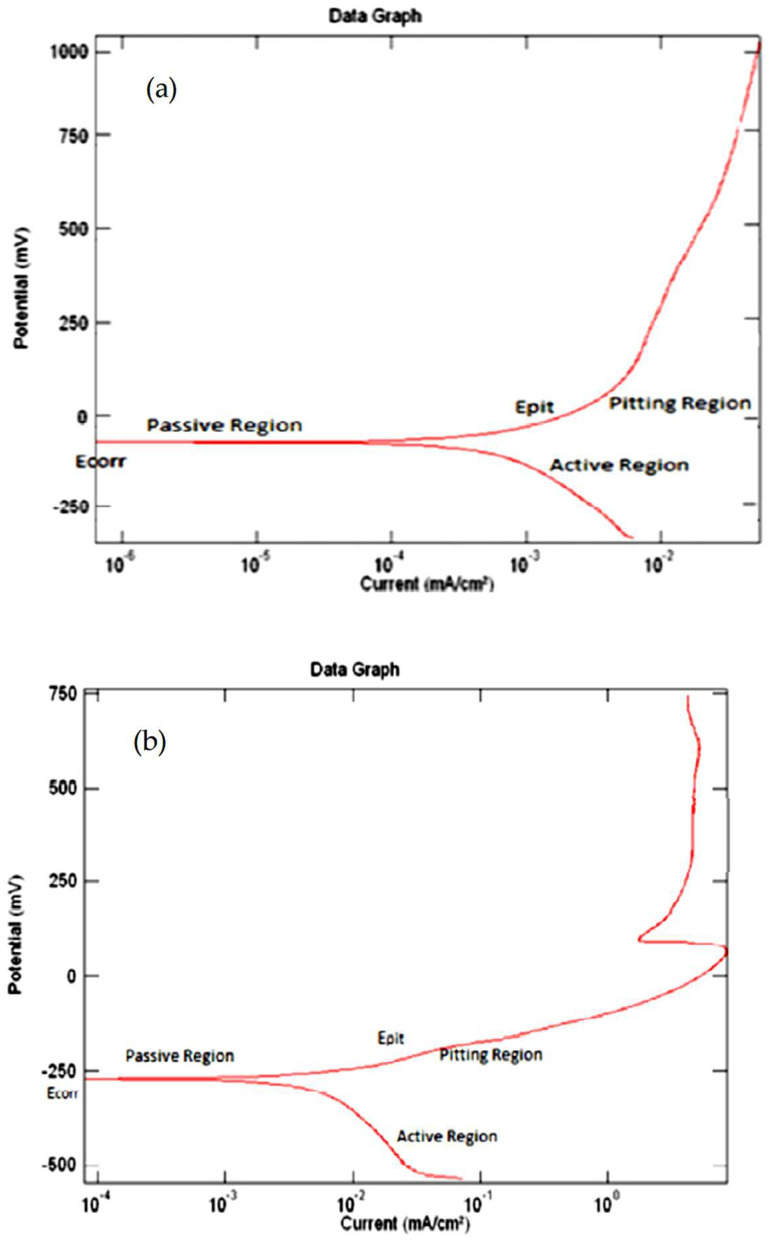
(**a**) Fresh water Tafel plot and (**b**) Hank’s solution Tafel plot [21] (reused with permission from Elsevier).

The Tafel plot revealed both active and passive characterization of the test sample that was exposed to an aggressive aqueous environment as shown in Figure 10. In both situations, it was found that Hank’s solution had a higher environment-induced degradation or corrosion potential than fresh water [39]. The current density of the coated specimen was found to decrease in the anodic zone and increase in the passive zone. The coated specimens had a larger pitting potential than the uncoated test specimens. The coating essentially shielded the metal from localized degradation caused by the environment [40].

In comparison with Hank’s solution, fresh water showed a lower rate for environment-induced degradation of the chosen copper-aluminum-beryllium shape memory alloy (SMA). The resistance to environment-induced degradation, or corrosion, of the copper-aluminum-beryllium shape memory alloy was improved by the presence of a titanium coating [41]. It was assumed that the occurrence of environment-induced degradation, or corrosion, would tend to alter the innate shape memory characteristics of the chosen alloy since it tended to alter the composition of the alloy. Therefore, this feature needs to be carefully considered when selecting this alloy for a given application [42,43,44]. The titanium dioxide (TiO_2_) layer formed as a passive layer on the surface of the alloy served as a barrier and slowed the process of degradation or corrosion induced by the aggressive environment. The covering tended to slow the occurrence of “pitting” corrosion. When compared with a shape memory alloy (SMA) that was not coated, current density was lower for the coated shape memory alloy (SMA) [45,46,47].

## 5. Cu-Al-Be-Mn Tetrad Memory Alloys

The copper-aluminum-beryllium-manganese quaternary alloy has unique mechanical properties to include the following:

(a) Excellent shape remembrance;
(b) Appreciable mechanical strength;
(c) Enough immerge ability due to martensitic transfiguration and pseudo-elasticity;
(d) Speculative uniqueness to absorb sound, vibrations, and mechanical waves due to coarse grain.

The usage of shape memory alloys (SMAs) has been extremely successful in biomedical applications due essentially to the functionality properties of the alloys, which improve overall effectiveness, coupled with a potential for less invasive surgeries. Its biocompatibility is its primary biomedical application. Another significant area for the application of shape memory alloys (SMAs) is the domain of medicine, where pseudo-elasticity is effectively used to identify various components to include the following [48]:

(a) Filters for embolic protection;
(b) Tooth aligning wires;
(c) Cardiovascular stents;
(d) Microsurgical and endoscopic devices.

**Figure 11:**
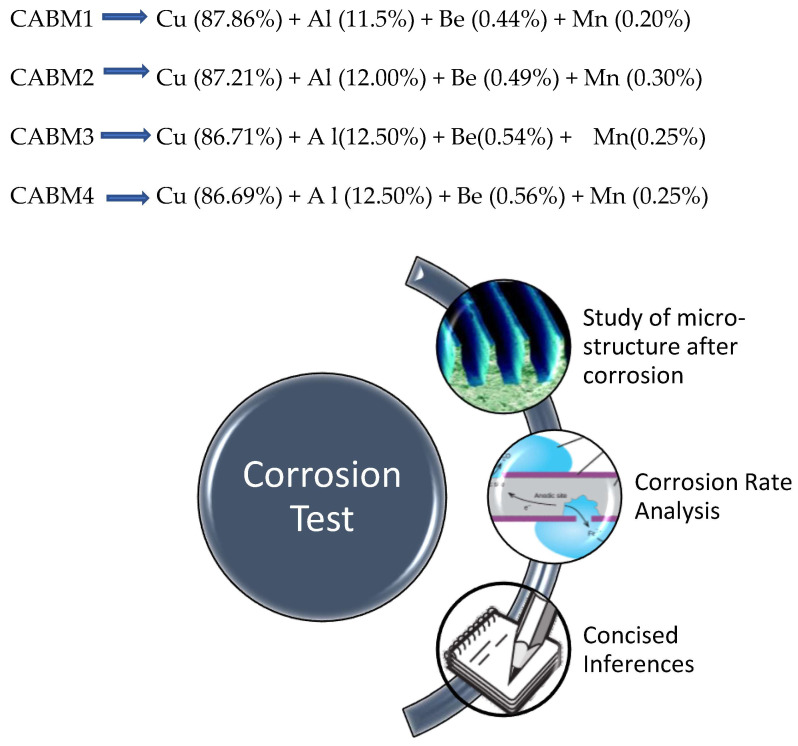
Skeletal structure of the Cu-Al-Be-Mn tetrad shape memory alloy.

Let us consider four different copper-aluminum-beryllium–manganese (CABM) alloys having different chemical compositions as shown in Figure 11.

### 5.1. Test Procedure for Evaluating the Influence of Environment

The created shape memory alloy (SMA) was pieced into a plane that was square in shape and measured 20.00 mm × 20.00 mm × 1.00 mm. The plane was then polished using emery paper of various sizes before being dusted with alumina (Al_2_O_3_) powder. The microstructure of the polished sample was examined before the test specimen was exposed to the three different aqueous environments, namely [49]:

(i) Ocean water (H_2_O);
(ii) Fresh water (H_2_O);
(iii) Hank’s solution.

**Figure 12:**
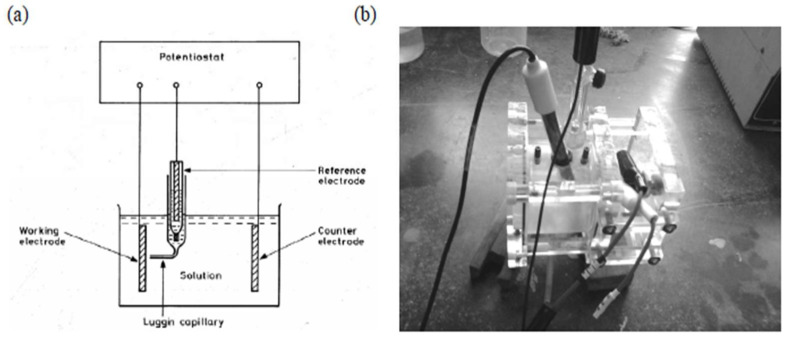
(**a**) Schematic representation of electrochemical cell. (**b**) Setup of electrochemical testing [50] (reused with permission from Elsevier).

A pictorial representation of an electrochemical cell is shown in Figure 12a. As shown in the figure, 150 mL of the desired solution was taken for the standard test cell (Figure 12b). After positioning of the sample and electrodes in the proper locations, an area of 0.16 cm^2^ was exposed to the aqueous solution. The standard calomel electrode, platinum electrode, and specimen electrode were preserved in their appropriate holders and linked to a potentiostat. Data specific to both voltage and current were collected using the Princeton applied research corrosion measurement program. The test data were displayed as the variation of voltage (E) versus current (I). A careful study and observation of the microstructure of the sample was made following exposure to an aqueous environment and the resultant environment-induced degradation, or corrosion, and the corrosion potentials E_corr_ and I_corr_ were established.

### 5.2. Results

#### 5.2.1. Study of Microstructure Following Exposure to Chosen Environment

**Figure 13:**
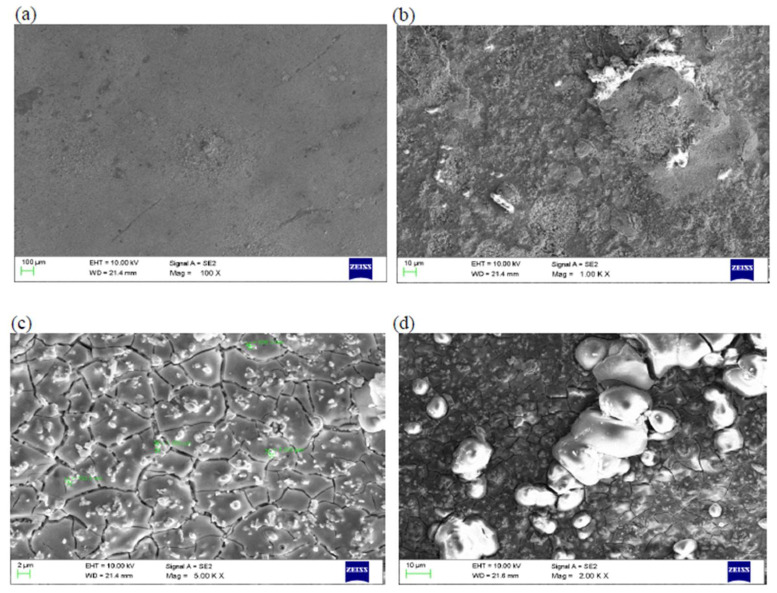
(**a**) CABM4 sample before corrosion. (**b**) CABM4 sample upon exposure to fresh water. (**c**) CABM4 sample after exposure to sea water. (**d**) CABM4 sample after exposure to Hank’s solution [50] (reused with permission from Elsevier).

The microstructure does exert a significant impact on both mechanical properties and physical properties, which has an influence on how these materials are chosen and used. Even though the alloys exhibit the austenitic parent phase upon casting, following step quenching, a complete change from austenite to lath martensite occurs. Step quenching demonstrates the entire transformation of austenite to martensite without a precipitate being formed [51]. Both shape memory effect and pseudo-elastic effect, as seen from the viewpoint of the microstructure, are outcomes of a solid phase microstructure transformation from austenite to martensite that could be triggered using a healthy synergism of mechanical pressure and thermal pressure. The chosen test piece was dried in laboratory air following exposure to an aggressive aqueous environment. Then, its microstructure was carefully examined. The micromorphology of the exposed and degraded test specimens of the copper-aluminum-beryllium–manganese sample (CABM4) in both sea water and Hank’s solution are shown in Figure 13c,d. The micromorphology of the exposed and degraded test specimens of the CABM4 mixture following exposure to fresh water (H_2_O) is shown in Figure 13b. It is clear from a comparison of the three microstructures that the environment of fresh water caused very little degradation by way of corrosion to the chosen sample. It was observed that the degradation that was experienced upon exposure to sea or marine water was much more complex than exposure of the test specimens to Hank’s solution (Figure 13d). The pits resulting from environment-induced degradation, or corrosion, that were apparent in the microstructure are the dark patches [52].

#### 5.2.2. Shape Memory Effect

**Figure 14:**
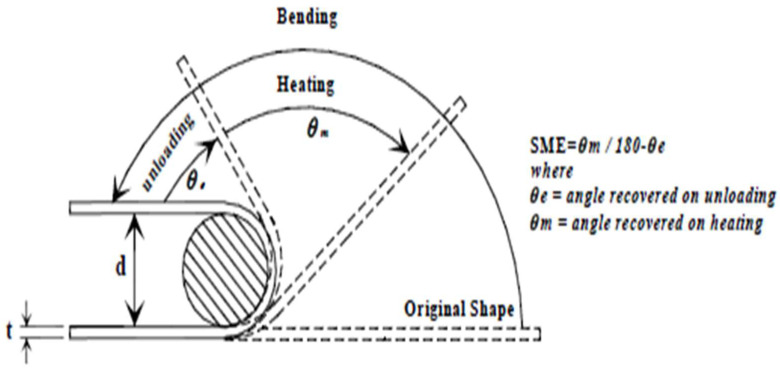
Bending test to find strain restoration [50] (reused with permission from Elsevier).

The bend test, as shown in Figure 14, was used to assess the shape memory effect (SME) of the chosen alloy. The results are presented in Table 2. The formula used for the calculation of the shape memory effect is:

**Percentage of Shape Memory Effect = θ_m_/(180 − θ_e_)**

#### 5.2.3. Analysis of Rate of Degradation Due to the Environment

A metallurgical corrosion analyzer system gill AC and an electrochemical corrosion cell were used to calculate both the rate of environment-induced degradation, or corrosive rate, and potential. E_corr_ was the actual location on the surface of the chosen material when the passive layer began to deplete [50]. It was seen that fresh water did not form pits. Additionally, it was noted that the rate of environment-induced degradation, i.e., the values of I_corr_, and E_corr_, were greater upon exposure to aqueous Hank’s solution than the sea (marine) water solution. Results revealed the resistance to environment-induced degradation, or corrosion, to increase with an increase in beryllium content (weight percent) in the alloy.

##### The Tafel Plots

Tafel plots were able to provide a precise measurement of corrosion current that varied inversely with the rate of environment-induced degradation, or corrosion. When compared with the weight reduction methods, this process was quick. The Tafel plots were graphed with potential (E) along the Y-axis and current (I) along a logarithmic X-axis. Five regions made up the curve, as follows [53,54]:

(i) The passivation region.
(ii) The elementary passive zone.
(iii) The initiation of passivation.
(iv) The active region.
(v) The trans-passivation zone.

**Figure 15:**
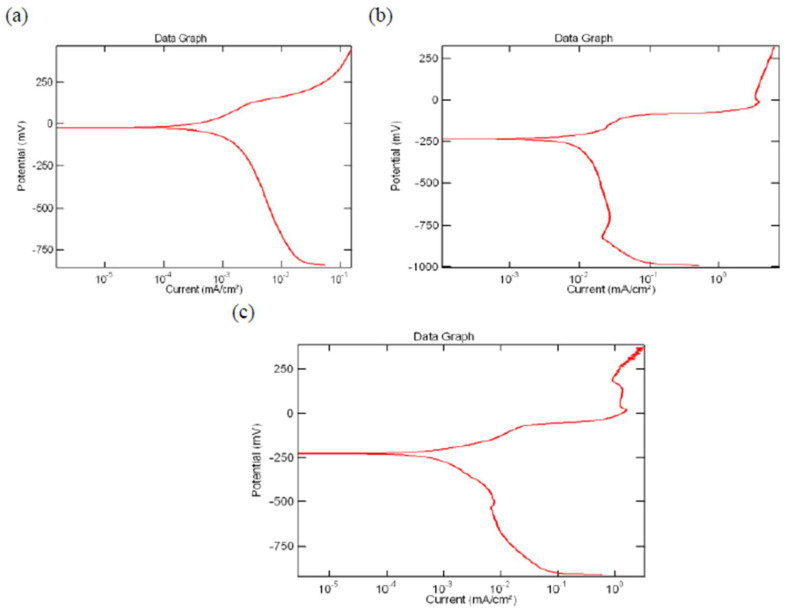
(**a**) Fresh water (H_2_O) PDC for the CABM4 alloy. (**b**) Ocean water (H_2_O) PDC for the CABM4 alloy. (**c**) Hank’s solution PDC for the CABM4 alloy. Ref. [50]. (Reused with permission from Elsevier).

It took some trial and error to determine the initial potential. The potential once established was maintained for the entire family of chosen alloys. The starting potential and reverse potential both fell between −250 mV and +800 mV. The sweep rate was kept at 5 mV/s [55]. The potentiodynamic plot for the CABM4 alloy in pure water (H_2_O), ocean (marine) water (H_2_O), and Hank’s solution is shown in Figure 15.

#### 5.2.4. Observations

Using the ingot metallurgy technique, the copper-aluminum-beryllium–manganese shape memory alloy (SMA) mixtures were created. The shape memory effect (SME) of the alloys was good. The entire transition from the austenite phase to the martensite phase in these alloys resulted in a positive shape memory effect (SME) [56]. The results of our study revealed the following:

1. Exposure to freshwater resulted in a lower rate of corrosion for the copper-aluminum-beryllium–manganese shape memory alloy (SMA) when compared with Hank’s solution and ocean water.
2. Hank’s solution had a stronger resistance to environment-induced degradation on the copper-aluminum-beryllium–manganese shape memory alloys (SMAs) than ocean water.
3. By adding trace amounts of beryllium to the alloy, the copper-aluminum-beryllium–manganese quaternary shape memory alloys (SMAs) revealed an improved resistance to degradation induced by the aqueous environment.
4. The Cu-Al-Be-Mn alloy had a remarkable 88 percent shape memory effect (SME).

## 6. The Cu-Al-Ni-xCo Shape Memory Alloys Biformed with Low-Carbon Steel

This alloy has a high damping capability greater than nitinol; in recent years, it has become the most sought-after shape memory alloy (SMA). The Cu-Al-Ni alloy is appropriate for applications spanning fasteners, buildings, bridge-cushioning components, oil well extraction, microscopic elements, and structures. However, damage caused by the conjoint and mutually interactive influences of degradation resulting from exposure to an aggressive environment and erosion should be taken into consideration when put to use in applications that are often exposed to aggressive aqueous environments, such as in coastal areas and even oil rigs [57]. In addition, using the shape memory alloy mixture as an element but not as an entire system is both feasible and cost-effective. However, because the chosen shape memory alloy is often in touch with other metals, the tendency for galvanic corrosion to occur is favored. The Cu-Al-Ni alloy is one of the demanding shape memory alloy candidates for the purpose of industrial use, primarily because it offers good resistance to environment-induced degradation due in essence to the presence of an alumina layer and its contributing role as a passive film.

It is crucial to keep in mind that the Cu-Al-Ni alloy has the drawback in that it is susceptible to post-quench ageing, which does affect and/or influence its mechanical properties upon sustained exposure to high-temperature service conditions that tend to gradually worsen with time. Accordingly, extensive research has been carried out to enhance the characteristics of the copper-aluminum-nickel alloy in order to both enable and expand its use in space applications while concurrently satisfying industrial needs and requirements.

In recent years, numerous methods have been used to improve the properties of the shape memory alloys (SMAs). One such method is a refinement in the grain size. In this method, quaternary elements such as zirconium and titanium are added in small quantities or trace amounts to the shape memory alloy. During the additions of elements such as titanium (Ti) and manganese (Mn), the resistance to environment-induced degradation of the copper-aluminum-nickel shape memory alloy gradually increases when the grain size decreases. On the opposite end, it has been claimed that the addition of cobalt to a copper-aluminum-nickel alloy results in both an improvement in mechanical properties and the temperature at which the austenite phase transforms. This, thereby, allows for the use of this combination at higher temperatures [58,59,60,61]. The Cu-Al-Ni shape memory alloy is less appealing than the popular Ni-Ti alloy, although offering relatively good mechanical properties and acceptable resistance to environment-induced degradation or corrosion, which places a restriction on its utilization. By the addition of quaternary elements, such as cobalt (Co), titanium (Ti), and manganese (Mn), in different proportions and subjecting the alloy to heat treatment, researchers have been able to improve the mechanical properties of the copper-aluminum-nickel alloy. The capability of the alloy for selection and use at higher temperatures is made possible by the addition of cobalt, although this fact does not improve its applicability [62,63,64,65,66]. Additional research studies on environment influences on the behavior of the copper-aluminum-nickel shape memory alloys under these conditions is needed primarily because its applications may often require an exposure to aggressive aqueous environments.

### 6.1. Electrochemical Test on Sample Prepared

**Figure 16:**
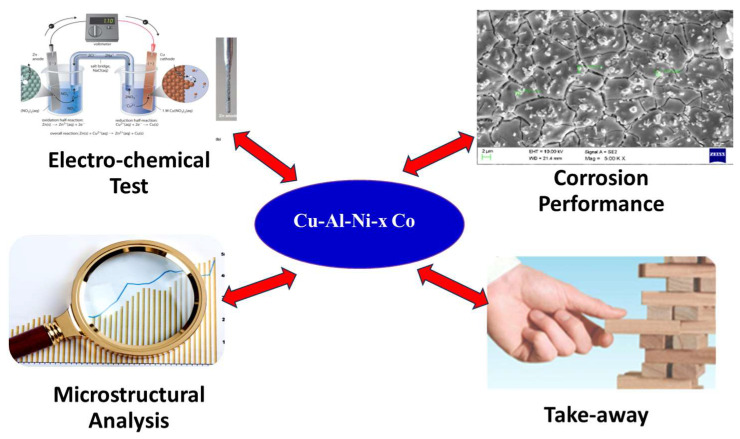
Corrosion study of the Cu-Al-Ni-xCo shape memory alloy.

The Tafel electrochemical test was used to investigate both the corrosive behavior and the working ability of the copper-aluminum-nickel alloy with the addition of cobalt and without the addition of cobalt as the fourth alloying element, i.e., the alloys (Figure 16).

(i) Copper-aluminum-nickel.
(ii) Copper-aluminum-nickel-1.0 wt% Co.
(iii) Copper-aluminum-nickel-0.4 wt% Co.

The test samples were coupled with low-carbon steel based on electrochemical measurements. The Cu-Al-Ni-(x) Co (x = 0 weight percent, 0.4 weight percent, and 1 weight percent) shape memory alloy (SMA) ingot was cut into tiny pieces that had dimensions of 25.00 mm (L) × 20.00 mm (W) × 2.00 mm (t) in order to make it both suitable and appropriate for the electrochemical test [67,68,69,70,71]. The samples were made from the as-homogenized ingots. The low-carbon steel bar was divided into pieces. Each piece measured 100.0 mm in length and 5.0 mm in width. Using epoxy, steel rebar and the biformed Cu-Al-Ni alloy were pieced or joined together. The Cu-Al-Ni-(x) Co shape memory alloys (SMAs) were also connected using copper wires. The samples were initially drilled and wires made of copper were then fastened to them using nuts and bolts. A fixed surface area of the rectangular test specimen was then subject to electrochemical tests. The tests were conducted using both a PARSTAT 2263 potentiostat and galvanostat at 20 °C in an amorphous glass cell that contained 325 milliliters of 3.50% sodium chloride solution. Potentiodynamic polarization studies were conducted using a three-electrode cell; a saturated calomel electrode (SCE) was used as the relating electrode [72]. The operating electrode chosen was the test specimen, while the counter electrode was a graphite rod. The start of each experiment was 225 mV below the open circuit voltage difference and the scan rate was maintained constant at 0.5 mV/s. Although the program permitted human control, fitting of the gathered test data was challenging. The Tafel plots were performed by choosing a section of the deterioration potential (φ_corr_) versus current density (I_corr_) curve and then estimating the potential value of the corrosion potential (φ_corr_) [73]. Every potential pointed to the saturated calomel electrode (SCE); the test data reported were the mean values after considering the standard deviation. To ensure repeatability, each experiment was repeated three times.

### 6.2. Microstructural Analysis

Two separate phases were seen, namely (i) the plate-like γ and (ii) the needle-like β. One other independent study has also identified these two phases [74]. The plate-like γ phase essentially had a 2H structure. The grain size of the chosen and studied copper-aluminum-nickel shape memory alloys tended to grow larger as they aged. Furthermore, careful observations revealed that, with aging, the plate thickness of the γ phase increased while that of the needle-like β phase decreased. The peak became stronger with aging, which could be correlated with coarsening of the martensite phase. No other phases or peaks were found following the ageing treatment, based on X-ray diffraction data. At the opposite end, the electron micrographs clearly revealed that as the cobalt content in the alloy increased, the grain size of the copper-aluminum-nickel-(x) cobalt shape memory alloy decreased and a new phase, i.e., precipitate, developed in the matrix. The precipitate existed both at and along the grain boundary regions and gradually grew larger in size with an increase in cobalt content in the alloy. The energy dispersive X analysis (EDAX) revealed the precipitate to have a high cobalt concentration. Grain growth occurred upon aging of the –copper-aluminum-nickel-1.0 weight percent cobalt shape memory alloy, just as it did for the copper-aluminum-nickel alloy without the addition of cobalt. There was a noticeable difference in microstructure of the copper-aluminum-nickel-1.0 wt.% Co alloy both before the aging treatment and after the aging treatment. For the copper-aluminum-nickel-0.4 wt.% Co alloy and the copper-aluminum-nickel-1.0 wt.% Co alloy, the Al_75_Co_22_Ni_3_ precipitates were both non-uniform in size and non-uniform in distribution prior to aging. Some of the precipitates had a diameter of 0.3 μm, while others had a diameter of only 0.1 micrometer. The smaller precipitates gradually disintegrated following the aging treatment, leaving behind only the larger precipitates, whose sizes had shrunk to about 0.25 μm [75]. The X-ray diffraction data were significantly impacted by both dissolution and size reduction of the Al_75_Co_22_Ni_3_ precipitates. The peak intensity of the copper-aluminum-nickel-1.0 weight percent cobalt alloy revealed a noticeable decrease with ageing. However, after aging, the peak for the Al_75_Co_22_Ni_3_ precipitate in the copper-aluminum-nickel-0.4 weight percent cobalt alloy increased [76]. This outcome was explained using the logic that the copper-aluminum-nickel-0.4 weight percent cobalt shape memory alloy (SMA) was still in the early stages of precipitate evolution.

### 6.3. Performance upon Exposure to an Aqueous Environment

After 40 min of exposure to an aqueous environment, the polarization curves for both the aged Cu-Al-Ni alloy and unaged copper-aluminum-nickel alloy biformed with low-carbon steel rebar and isolated with the low-carbon steel rebar were determined from electrochemical experiments using a 3.50 percent sodium chloride solution. The corrosion potential for the linked copper-aluminum-nickel/low-carbon steel was 313.78 mV, which was between the corrosion potentials of isolated copper-aluminum-nickel alloy (18.025 millivolt) and low-carbon steel (422.521 millivolt). However, the connected material’s current density, which measured the rate of environment-induced degradation, gradually rose to 57.42 A/cm^2^, thereby exceeding the current density of the two chosen materials, which was 19.231 A/cm^2^ for the low-carbon steel and 28.592 A/cm^2^ for the copper-aluminum-nickel alloy. Galvanic corrosion, which was favored to occur when the two distinct metals came into contact, was to blame for this event [77]. On the other hand, aging treatment had a substantial influence on environment-induced degradation behavior of the linked Cu-Al-Ni and low-carbon steel sample. Degradation-induced by the environment on the copper-aluminum-nickel alloy slowed down, following ageing treatment for both the coupled samples and the uncoupled samples. This outcome was explained by coarsening of the β phase during aging. The presence of aluminum in plate-like morphology may have made the passive film on the surface to be stable and thereby encouraged the formation and presence of layers of alumina (Al_2_O_3_) [78]. Additionally, the surface area diminished microscopically as the needle-like β phase in the alloy shrank. According to the test data, it was found that the rate of environment-induced degradation decreased as the level of cobalt increased. In a different way, with the addition of cobalt, the resistance offered to environment-induced degradation of the Cu-Al-Ni alloy improved. This feature could be attributed to the role and contribution of cobalt addition in refining the grain size. There have been few discoveries that have shown a smaller grain size and large volume percentage of the precipitates to improve both the compactness and stableness of the passive layer leading to an overall improvement in the shape memory alloys’ (SMAs) resistance to environment-induced degradation or corrosion [79]. The trends shown by the connected samples mirrored those of the uncoupled samples. It was discovered that the copper-aluminum-nickel-0.4 wt percent cobalt alloy revealed an actual drop in the corrosion rate after ageing, which could be attributed to microstructural changes as a direct consequence of both ageing and the making of the alloy. For the case of the copper-aluminum-nickel 0.4 wt% cobalt alloy, the precipitate’s volume fraction was at the initiation stage [80]. However, with the aging of the sample, the density of the fine precipitates gradually improved. The evolution of precipitates contributed to increasing the passive film’s stability and overall compactness. Additionally, the size and dispersion of the precipitates had an impact on enhancing and/or improving the resistance to environment-induced degradation or corrosion. The corrosion resistance of the copper-aluminum-nickel-1.0 weight percent cobalt shape memory alloy (SMA), in which precipitation was numerous, was improved both by a noticeable reduction in size and an increase in homogeneity of the precipitates.

Additionally, a grain boundary, or other location, where the precipitates were separated often resulted in a change in the substrate. An equal anode–cathode dispersion would tend to decrease the localized galvanic corrosion between the matrix and the fine precipitates.

### 6.4. Interpretation on the Experimental Findings

1. Ageing treatment of the copper-aluminum-nickel alloy with the addition of cobalt as the fourth element promoted grain refinement and produced fine precipitates of the Al_75_Co_22_Ni_3_ phase that contributed to increasing both the compactness and stability due to the formation and presence of a passive film and thereby improved the corrosive resistance.
2. After being adjusted with the addition of 1 weight percent of cobalt along with ageing treatment, the linked copper-aluminum-nickel and low-carbon steel shape memory alloy (SMA) revealed an optimum value of corrosion resistance. This resulted in reducing the rate of environment-induced degradation, or corrosion, by well over 50 percent when compared with the low-carbon steel sample (uncoupled).
3. After the addition of 1 weight percent of cobalt and ageing at 250 °C for 48 h, the copper-aluminum-nickel shape memory alloy revealed a microhardness of 340 Hv.

## 7. The Cu-Zn-Al Shape Memory Alloy in Monitored Ambience

Copper–zinc–aluminum alloys are often chosen for use in a broad scope of engineering fields, primarily because of a combination of unique properties that makes them simple to produce. The copper-based alloy has been extensively studied in a variety of environments, but additional research is still needed to fully understand how this alloy reacts upon exposure to an aggressive environment and the resultant degradation induced by the environment, i.e., corrosion [81]. Centrifugal casting was used to create the copper alloy, which contained 21% zinc and 5% aluminum.

Using a combination of induction melting and centrifugal casting, the CuZnAl shape memory alloy (SMA) was created. With the help of a ceramic crucible made of vitreous silica and zirconium dioxide (ZrO2), pure metals, such as electrolytic copper (99.99%), aluminum 1100 alloy in sheet form (99.95%), and zinc pieces (99.9%), were easily melted in an induction furnace in an environment of argon and at a pressure below the ambient pressure. In a graphite mold, the molten alloy was centrifugally cast. Since an observable loss of zinc occurred during the melting process, the cast specimens were carefully monitored to determine both their composition and shape memory characteristics. If required, the cast materials were remelted along with the addition of pure metals to get a composition that was close to the nominal composition [82,83,84,85].

The as-cast specimens were analyzed to ascertain both their composition and microstructure. Intricacies specific to the microstructure were studied using the techniques of

(a) X-ray diffraction;
(b) Electronic microscopy;
(c) Optical microscopy after chemically etching the polished surfaces using a chemical reagent. The reagent used was ferric chloride (FeCl3) in hydrochloric acid (HCl) solution.

The potentiodynamic anodic polarization measurements were used to systematically analyze the environment-induced degradation behavior or response of the chosen shape memory alloy (SMA). This was performed using a thin plate of the chosen shape memory alloy (SMA) that was taken out of a cast bar using a diamond blade. This was followed by polishing the thin plate using silicon carbide (SiC) -impregnated emery paper (up to 600 mesh) and subsequently cleaning the polished surface using acetone. Thereafter, the ASTM G5-compliant corrosion test was conducted. About 2 square cm of the surface was exposed to an aggressive environment that favored the occurrence of degradation, referred to as corrosion. The working electrode surface was close to the reference electrode. The working electrode and reference electrode were separated by roughly 1 square cm from an auxiliary electrode, which was essentially a wire mesh made of platinum.

To simulate the various conditions prevailing in reality, a variety of test solutions were used and essentially included the following:

(a) 3.5% weight sodium chloride (NaCl) aqueous solution that simulated a marine environment.
(b) Acid solutions of 1 M, 0.1 M, and 0.01 M nitric acid (HNO_3_) that replicated acid rain in a metropolitan setting.
(c) Acid solutions of 1 M, 0.1 M, and 0.01 M sulfuric acid (H_2_SO_4_) that replicated acid rain in an industrial setting.

### The Test Results

The surroundings utilized in the experiment exerted an influence on the behavior of the test specimens used that were subsequently analyzed. Martensitic lamellae were an easy target for the onset of environment-induced degradation in an environment of acid solution. The passivation phenomena were not easily visible and the environment-induced degradation rates tended to increase as a function of solution concentration [86].

The chosen shape memory alloys exhibited a decreasing trend when tested in concentrated sulfuric acid (H_2_SO_4_) and sodium chloride (NaCl) solutions. The current density also decreased and stabilized at high imposed potentials. Although these processes resembled passivation, the observed reduction in environment-induced degradation or corrosion was caused by a gradual buildup of layers of corrosive products. The pores present on the test sample surface permitted the degradation caused by the aqueous environment to continue by allowing or permitting a gradual diffusion of the chemical species from the interface of the deteriorating metal to the mixture. This layer eventually ruptured when a high potential (above 1.2 V) was reached, which resulted in the occurrence of pitting [87,88]. All the test specimens preserved the shape memory effect after the potentiodynamic test. The conditions suitable for oxidation resulted in the presence of flaws close to the grain boundary. The presence of these flaws caused the material to fail by the initiation of fine microscopic voids and their gradual growth and eventual coalescence to form one or more fine microscopic cracks that tended to propagate along the grain boundaries.

## 8. Nature of Degradation When Cobalt Is Added to the Nickel–Titanium Shape Memory Alloy (SMA) in Normal Saline Solution

Due to their strong biocompatibility, resistance to degradation-induced by the aqueous environment, and a high elastic modulus, the NiTi alloys are often chosen for use in medical devices. In addition, the NiTi alloys containing 1–2 percent cobalt offer the following attributes:

(a) A 30 percent higher modulus than the NiTi alloys.
(b) A distinct loading plateau and an unloading plateau.
(c) Non-reactivity in two tests, namely hemolysis and cytotoxicity.

An enhancement in yield strength was made possible for the NiTi alloys with the addition of 2–10 percent cobalt [89,90,91]. Since the martensitic transition temperature occurred by separation of the R phase with the addition of cobalt, the results could be better explained by a two-step transformation process.

With the use of wire electro discharge machining (EDM), the rate of material removal, microstructure, and hardness were also investigated for the alloys containing up to 10% cobalt. The effect of cobalt on overall resistance to environment-induced degradation of the chosen alloys must be investigated prior to their selection for use in a specific application. According to Huang and co-workers, upon investigation of the environment-induced degradation characteristics of the chosen alloy on exposure to an aggressive aqueous environment, they behaved like the NiTi alloys that did not contain cobalt and were not adversely influenced by the occurrence of galvanic corrosion.

The investigations were conducted in a mild climate where degradation due to both pitting and crevice deterioration were not typical. It was anticipated that the addition of enough cobalt (Co) to an alloy would boost its resistance to this type of deterioration, primarily because the cobalt-base alloys tended to resist the localized effects of environment-induced degradation, unless fretting and other mechanical deterioration occurred on the surface oxide [92]. Our recent study on investigating and understanding the corrosion and electrochemical behavior of the cobalt-based magnetic shape memory alloys (MSMAs) in 0.50 M sodium chloride solution revealed the vital corrosion resistance of the MSMAs. This was well supported by the following:

(i) Electrochemical tests;
(ii) XPS;
(iii) Scanning electron microscopy (SEM) observations;
(iv) Energy dispersive X-ray (EDX) analysis.

**Figure 17:**
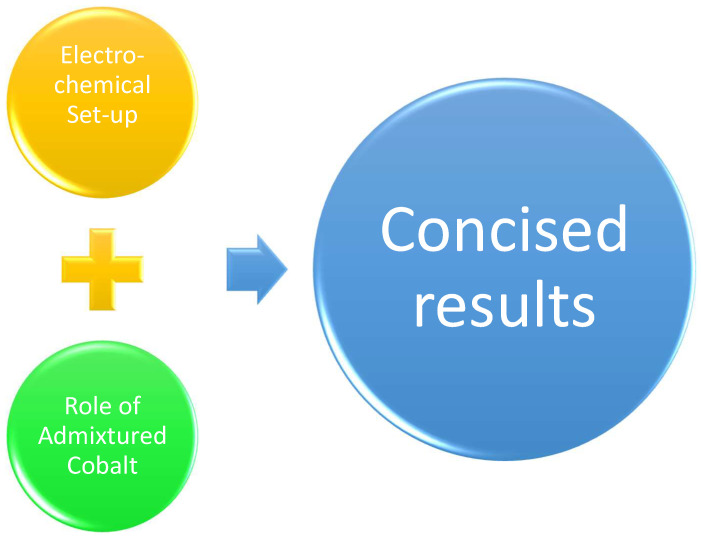
Study of the effect of cobalt added to nickel–titanium (NiTi).

Several studies on both the role and contribution of the addition of cobalt (Co) on the environment-induced degradation behavior of NiTi shape memory alloys (SMAs) are limited. To accomplish its goal, a recent study used a variety of electrochemical techniques in addition to (i) scanning electron microscopy (SEM) observations, (ii) energy dispersive X-ray (EDX) analysis, and (iii) full XPS studies as presented in Figure 17. In both uniform degradation and pitting degradation experienced by the chosen NiTi(x)Co (x = 14.0%, 1.50%, and 4.0%) shape memory alloy (SMA) upon its exposure to a 0.90% sodium chloride solution at 38 °C, to know the influence on passivation of the alloy containing cobalt (Co), the impact of cobalt addition on passivation translated into a clear improvement in the overall resistance to environment-induced degradation of the chosen alloy [93,94,95].

### 8.1. Electrochemical Setup and Solutions

Calomel saturated electrode and platinum (Pt), which is a mesh, electrodes were used as the reference electrode and auxiliary electrode in a typical packed and covered three-electrode cell for the purpose of conducting electrochemical studies. A potentiostat connected to a PC along with a galvanostat were attached to an electrochemical cell to both run and concurrently observe the electrochemical procedures used in the investigation. Measurements were made using a freshly made 0.9% normal saline (NaCl) solution that was purified prior to use [96]. Analytical grade salt, which was acquired from Sigma-Aldrich (New York, NY, USA), was used for the experiment.

Using a temperature-monitored and controlled water bath, with water continuously circulating through the outer packed cell jacket, the temperature of the solution was kept constant at 37 °C.

### 8.2. Role of Addition of Cobalt

#### Study of Microstructure

In addition to precipitation and presence of the intermetallic phase (Ti_2_Ni), it also included the parent phase (i.e., austenite) and the matrix phase (i.e., martensite). The addition of cobalt influenced the phase volume fraction (V_f_) observed in the microstructure. When the percentage of cobalt was increased from 0 to 1.5 atomic percent, the volume fraction (V_f_) of the parent phase (austenite) gradually decreased and even disappeared from the microstructure when the cobalt content was increased to 4 atomic percent. Additionally, density of the intermetallic phase (Ti_2_Ni) in the microstructure of the titanium–nickel shape memory alloy (SMA) decreased with the addition of cobalt. In the microstructure of the NiTiCo (0 atomic percent Co) alloy, precipitates of the Ti_2_Ni phase had a range of chemical composition. The nickel concentration was 34.15 atomic percent, while the titanium content in the Ti2Ni phase was 65.85 atomic percent. Additionally, as the level of cobalt content in the NiTi alloy increased, so did the cobalt content in the intermetallic phase (Ti_2_Ni) [97].

In addition, for the TiNi (with 0 atomic percent cobalt) shape memory alloy (SMA) the matrix phase, i.e., martensite, had a chemical composition that included both nickel and titanium. Based on the amount of cobalt added to the TiNi shape memory alloy (SMA), different percentages of nickel (Ni), titanium (Ti), and cobalt (Co) were found to be present in the martensite phase.

### 8.3. Summary of the Results

The microstructure of the studied shape memory alloys (SMAs) was affected by the addition and/or presence of cobalt by decreasing both the average size of the Ti_2_Ni intermetallic phase and the total area occupied by this phase. Cobalt affected overall resistance of the alloy to environment-induced degradation through a variety of mechanisms, including the following:

(a) Overall homogeneity of the surface electrochemical characteristics;
(b) Reduced activity of the microgalvanic cells.

With the addition of cobalt (Co), the number of chloride ions in the passive layer covering the surface of the chosen shape memory alloy (SMA) noticeably decreased. This demonstrated an overall stableness of the passive layer coupled with an improved resistance to the occurrence of both uniform environment-induced degradation or corrosion and pitting deterioration processes [98]. Despite this, the overall chemistry of the passive layer, which was made up of titanium dioxide (TiO_2_), did not significantly change.

## 9. The Nickel-Titanium Shape Memory Alloy Sintered by Spark Plasma Sintering (SPS)

### 9.1. Development of the Sintered Nickel-Titanium Shape Memory Alloy (SMA)

**Figure 18:**
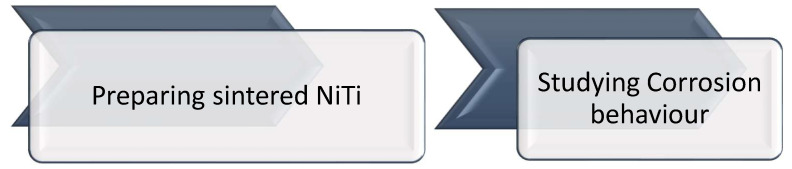
Corrosion behavior of the sintered nickel–titanium alloy.

To create 50-50 NiTi, nickel (average size 40 μm, 99.7% purity) and titanium (mean size 47 μm, 99.7% purity) powders were alloyed for 5 h in a planetary ball mill system at a high energy. The powder mixture was milled for a full 30 h in the same manner as before. The mixed powder could safely be classified to contain particles having an average size in the range 10 μm to 45 μm [98]. Both the milling parameters and process parameters chosen were essentially based on those used in an earlier study as shown in Figure 18. Since it exerted an influence on both size and uniformity of the powder particles, the duration of milling had a significant influence on alloying of the NiTi alloy.

### 9.2. Characteristics upon Exposure to an Aggressive Environment

The NiTi alloys were sintered using the spark plasma sintering (SPS) method and in a vacuum environment, resulting thereby in high densification. Investigations were conducted into environment-related activity of the sintered alloy. Additionally, an evaluation of both the microstructure and dispersion of the elements in the degraded region was made. Using the method of spark plasma sintering (SPS), high density NiTi alloys having particles of size 10 μm were sintered. When the specimen was exposed to plastic deformation at a given pressure together with electrical discharge at ambient pressure, the goal was to achieve an improvement in the density coupled with a smaller grain size and a slender grain morphology. While the NiTi alloys were created using particles having a size of 10.0 μm and the spark plasma sintering (SPS) method at 910 °C, it was fully consolidated and had a dendritic structure. The presence of secondary phases in the microstructure, such as NiTi_2_ and Ni_3_Ti, prevented the occurrence of environment-induced degradation or corrosion. Low-corrosion current density and high-corrosion potential were easily attained for the nickel–titanium alloys that were created at a maximum binding temperature by using smaller size particles. Increased polarization current density did have an impact on the rate of degradation induced by the aqueous environment. The rate of degradation did affect the weight loss experienced by the NiTi alloys [98,99]. Thus, for the NiTi alloys that were produced using 10 μm particles and at a high SPS temperature, the loss in weight was essentially governed by the polarization current density. Due to a homogeneous distribution of the corrosion-resistant phases coupled with a decrease in the polarization current density, the rate of degradation caused by exposure to an aggressive aqueous environment was lowered by as much as 74 percent [100]. For the case of the nickel–titanium shape memory alloys (SMAs), a fine size of the starting particles coupled with a high sintering temperature essentially created a pore-free skin surface. This surface was beneficial for improving the overall resistance of the alloy to degradation caused by the aggressive aqueous environment.

## 10. Biocompatibility of Shape Memory Alloys and Its Progress

Understanding the corrosion process is the key to understanding metal biodegradation. Metals are biodegraded via electrochemical corrosion in the physiologically slightly alkaline environment (pH 7.4), which is often made up of anodic and cathodic processes [100,101]. It has been discovered that anodic processes include both metal breakdown and electron release:

*M* − *ne*^−^ → *M^n^*^+^ (anodic reaction) (1)

The subsequent cathodic processes that take place in the electrolyte to capture these electrons result in the production of hydroxide ions.

*M^n^*^+^ + *n*OH^−^ → *M*(OH)*_n_* (corrosion product) (2)

In order to increase the overall corrosion rate of the alloy during the manipulation of biodegradation performance, it is essential to enhance the electrochemical corrosion effects happening between the SMA matrix and electrolytes or other phases. Structure, implantation duration, and SMA phase transition temperatures are additional variables that may impact SMA biodegradation efficacy [102]. Both macro- and microstructures have a significant impact on biodegradations. For instance, NiTi SMA is shown to have reduced corrosion resistance as porosity increases due to bigger pore openings, but considerably higher corrosion rates [103]. Biocompatibility of Ni-based shape memory alloys prove that they are more suitable for biomedical implants and pursue their highest degree of functionality in the applications they are involved in.

### 10.1. Methods to Improve Biocompatibility

#### 10.1.1. Grain Refinement

It has been generally documented that grain refining works well to slow the rate of corrosion. The SME and SE/PE of SMAs are not anticipated to be impacted by grain refining, since phase transitions are often not linked to this process. In addition, grain refining may eliminate any biocompatibility issues brought on by the inclusion of additional components and further increase the mechanical characteristics of SMAs based on the Hall–Petch connection [104]. The formation of a more cohesive and compact protective corrosion layer, the prevention of preferred crystallographic pitting restrained by increased grain boundaries, and the improvement of basal plane intensity can all be used to explain the reduction in corrosion rates caused by grain refinement.

#### 10.1.2. Surface Coatings

Additionally, Ni-based SMA’s medicinal uses may be expanded by surface coatings with additional bioactive or therapeutic properties, including antibacterial and drug-loading properties. It maintains the mechanical characteristics and shape memory behaviors of the underlying metal substrate without changing its microstructure [105]. To avoid abnormally low biodegradation rates, the coating layer should preferably be degraded or detached after the creation of the passivation layers. In situ conversion coating and ex situ deposition coating are two options for achieving the needed transitory corrosion protection. For the conversion coating, in situ interfacial interactions between the metal substrate and coating solution produce the noble coating layer. The native surface of the metal substrate might change into oxide or salts depending on the coating solution. Due to the in situ growth of the coating layer from the metal substrate, a suitable interfacial adhesion may be achieved to avoid premature coating layer detachment or potential penetration of corrosive media into the metal–coating layer interface [106].

### 10.2. Challenges in Employing Shape Memory Alloys at Bioapplications

(1) To fully comprehend the causes and mechanisms underlying temperature- or stress-induced phase changes between austenite and martensite, further microstructural insights into the mechanisms underlying SMA properties are needed. Understanding these mechanisms can help design SMA and manipulate corrosion with beneficial recommendations. Through in situ studies of SMA deformation and mechanical reactions during the phase transition, advanced microscopy is anticipated to play a significant role in this respect [106]. At the austenite–martensite contacts, crucial information may include the lattice resistance, steps, and dislocation arrays.
(2) These materials must be modified to be suited for more nuanced biological applications, which calls for precise control of the SMA transition temperature and stress. There is evidence that alloy composition and thermomechanical treatments can adjust SMA transition temperatures [107]. The difficulty in designing a functional device stem from the fact that such modulation is not precise enough to achieve the precise needed values. In order to overcome this issue, computational intelligence may be useful, as topological models, artificial neural networks, and Gaussian process regression may all be used to anticipate transformation temperature and stress.
(3) More research on passive films and film–metal interactions may provide new insights into the behavior of SMA corrosion. According to reports, TiO_2_ is crucial in preventing NiTi corrosion and the passive coatings on NiTi SMA display n-type semiconductor characteristics. While efficient TiO_2_ dissolution may be achieved by lowering the pH of the corrosion environment, doping levels can be enhanced by donor production at the metal–film interface to reduce the corrosion resistance of passive films [108]. Future work may focus on altering the chemical makeup of passive films to control their corrosion resistances or adjusting the doping levels of semiconducting passive films to produce metastable or stable pits and voids at the metal–film interface to speed up corrosion.

## 11. Conclusions

Based on a study aimed at understanding the effects of alloying and environment on the degradation response or corrosion behavior of shape memory alloys, the key findings are as follows:

1. The trend towards the use of less invasive techniques and microscopic applications will continue.
2. Processing capabilities are being noticeably improved and the shape memory alloys (SMAs) are gradually gaining for themselves a dominant place for due consideration by all engineers for selection and use in medical design-related applications and even technologies specific to emerging smart materials.
3. An increase in the selection and use of the shape memory alloys (SMAs) both in medicine and sensor technology can be expected. The shape memory alloys are currently being chosen for use in critical environments, such as high temperature vital fluids, i.e., the blood stream.
4. The nature of environment-induced degradation, or corrosion, of the shape memory alloys (SMAs) is presented and examined in this paper based on results obtained from tests conducted in aggressive aqueous environments.
5. Improving the resistance to environment-induced degradation of the shape memory alloys (SMAs) will pave the way for their selection and use in a sizeable number of applications. Further, discovering ways to resist degradation induced by the environment, through the development of passive films and coatings is both essential and desirable.

## Figures and Tables

**Table 1:** Shape memory effect (%) exhibited after the bend test [21] (reused with permission from Elsevier).

Sample	Diameter	Thickness	Angle Recovered	SME%
CAB1	32 mm	1	72	81
CAB2	32 mm	1	80	88
CAB3	32 mm	1	60	65
CAB4	32 mm	1	66	75
CAB1 → Cu (88.01%) + Al (11.5%) + Be (0.44%) [Without coating] CAB2 → Cu (88.05%) + Al (11.5%) + Be (0.45%) [Without coating] CAB3 → Cu (88.01%) + Al (11.5%) + Be (0.44%) [With coating] CAB4 → Cu (88.05%) + Al (11.5%) + Be (0.45%) [With coating]				

**Table 2:** Shape memory effect (SME) of the copper-aluminum-beryllium-manganese shape memory alloy (SMA). Ref. [50] (reused with permission from Elsevier).

Sample	Diameter	Thickness	Angle Recovered	SME%
CABM1	32 mm	1	155	82.39
CABM2	32 mm	1	148	77.93
CABM3	32 mm	1	157	83.57
CABM4	32 mm	1	163	88.78

## Data Availability

Data sharing not applicable. No new data were created or analyzed in this study. Data sharing is not applicable to this article.
